# Marrow Stromal Cell Infusion Rescues Hematopoiesis in Lethally Irradiated Mice despite Rapid Clearance after Infusion

**DOI:** 10.1155/2012/142530

**Published:** 2012-02-16

**Authors:** Xiaodong Yang, Ilango Balakrishnan, Beverly Torok-Storb, Manoj M. Pillai

**Affiliations:** ^1^Department of Medicine, University of Colorado Denver, Aurora, CO 80045, USA; ^2^Clinical Research Division, Fred Hutchinson Cancer Research Centre, Seattle, WA 98109, USA

## Abstract

Marrow stromal cells (MSCs, also termed mesenchymal stem cells) have been proposed as a promising cellular therapy for tissue injury including radiation-induced marrow failure, but evidence for a direct effect is lacking. To assess the effects of MSCs on survival after lethal irradiation, we infused syngeneic MSCs (either as immortalized MSCs clones or primary MSCs) intravenously into wild-type C57/Bl6 mice within 24 hours of lethal total body irradiation (TBI). Mice receiving either of the MSC preparations had significantly improved survival when compared to controls. In vivo imaging, immune histochemistry, and RT-PCR employed to detect MSCs indicated that the infused MSCs were predominantly localized to the lungs and rapidly cleared following infusion. Our results suggest that a single infusion of MSCs can improve survival after otherwise lethal TBI but the effect is not due to a direct interaction with, or contribution to, the damaged marrow by MSCs.

## 1. Introduction

High-dose ionizing radiation causes damage to many organs, especially those with highly proliferative cells such as the bone marrow and the gastrointestinal tract [1, 2]. Bone marrow failure is often the cause of death following moderate-to-severe exposures to radiation [3]. Several pharmacologic agents especially cytokines such as granulocyte colony stimulating factor (GCSF), granulocyte monocyte colony stimulating factor (GMCSF), interleukin 3 (IL3), and thrombopoietin (TPO) have been shown in various experimental models to mitigate hematopoietic effects of radiation and are approved for clinical use in scenarios of radiation-induced aplasia [4–6]. The efficacy of these agents is, however, limited to intermediate dose ranges which do not result in complete marrow ablation. Exposure to higher doses that result in complete marrow ablation requires the transplantation of a new lympho-hematopoietic system from a suitable donor [7]. As such transplantations are impractical to be performed expeditiously following exposure to high-dose radiation (accidental or nuclear attacks), there is significant interest in improving the efficacy of pharmacological agents as mentioned above and explore novel agents with efficacy at doses higher than what cytokines are typically effective. “Off-the shelf” cellular therapies that can be expanded from a few initial cells, frozen, and thawed for quick infusion and do not require extensive tissue-matching have been explored as alternatives to full allogeneic stem cell transplantation following such radiation exposures. Marrow stromal cells (MSCs, also referred to as mesenchymal stem cells) have been proposed as one such cellular therapy to aid regeneration of radiation-induced aplasia; MSCs have shown promise in preclinical studies in rodents and uncontrolled human trials to aid in the regeneration of damaged tissues in experimental models simulating acute graft versus graft disease (aGVHD) [8], renal failure [9], diabetes mellitus [10], and myocardial infarction [11]. However, despite enormous interest in using MSCs to aid in hematopoietic regeneration following radiation exposure, the benefit of MSC infusion on survival after radiation induced marrow damage has only been addressed by a few recent studies [12, 13]. In this study, we sought to determine if a single dose of MSC (either cloned or primary MSC cultures) following lethal dose irradiation would improve survival in the murine model. We also determined the spatial and temporal distribution of infused MSCs in recipients to help better understand the mechanism of action of these cells in improving hematopoietic reconstitution.

## 2. Methods

### 2.1. Marrow Stromal Cells

 Murine MSC lines were isolated by transducing a primary long-term culture of murine bone marrow with the LXSN-16 E6E7 retrovirus (encoding human papilloma viruses E6 and E7) followed by selection in G418 and ring-cloning as previously described [14]. Five MSC lines (denoted B6M1, 6, 7, 9, and 11) were used for further studies mixed in equal ratio. Primary MSCs were prepared by plating whole marrow mononuclear cells from adult female mice in DMEM supplemented with 10% fetal bovine serum, cultured till confluence, and expanded 3-4 passages. Both the cloned and primary MSCs were analyzed for ability to differentiate to osteoblastic, chondroblastic by utilizing commercial kits (Invitrogen, Carlsbad, CA) and adipocytic lineage by previously described techniques [15] (Supplementary Methods and Supplementary Figure 1 available online at doi:10.1155/2012/142530).

### 2.2. Animals and MSC Infusion

 All animal studies were approved by the University of Colorado Denver's animal care and use committee (IACUC). Female C57/Bl6 mice (Jackson Laboratories, Bar Harbor, ME) 6 to 8 weeks of age were used as recipients of MSC infusion. MSCs were suspended in 100 *μ*L of PBS (phosphate buffered saline) for infusion. Radiation was delivered using an X-ray irradiator (RS2000, Rad Source Technologies, Suwanee, GA) in two split doses. Drinking water was supplemented with neomycin (150 mg/L) and bacitracin (5 mg/L) to minimize infections [16]. Single MSC infusion was performed by tail vein injection from 16 to 24 hours after radiation. All animals were monitored for at least 7 weeks to document survival from radiation. Animals were monitored closely for evidence of distress resulting from hematopoietic failure (weight loss of over 15% of baseline, signs of infection, decreased feeding or activity) and were euthanized if deemed in irreversible distress.

### 2.3. Bioluminescent Imaging (BLI or In Vivo Imaging) of Labeled MSCs

 BLI was performed using Xenogen IVIS 200 system, MSCs labeled with fire fly luciferin (ffluc) were infused as described above and imaged at 4, 7, 24, and 72 hours after MSC infusion by intraperitoneal injection of luciferin and visualization as previously described [16] and detailed in Supplementary Methods.

### 2.4. Immune Histochemistry (IHC) and Quantitative Real-Time PCR (qRT PCR) for Firefly Luciferase (Ffluc)

 IHC was performed with mouse anti-ffluc antibody or isotype control (Novus biological) per published protocols on formalin-fixed paraffin embedded tissues harvested at days 4 or 7 after infusion of labeled MSCs [17]. Total RNA was extracted from tissues at same time points and quantitative real-time PCR was performed on cDNA prepared from RNA with primers specific to ffluc or housekeeping gene TBP (Supplementary Methods).

### 2.5. Statistical Analyses

Kaplan Meir survival curves were constructed and *P* values calculated using MedCalc Software (Mariakerke, Belgium).

## 3. Results and Discussion

### 3.1. Single Infusion of MSCs Significantly Improves Survival in Lethally Irradiated Mice

**Figure 1:**
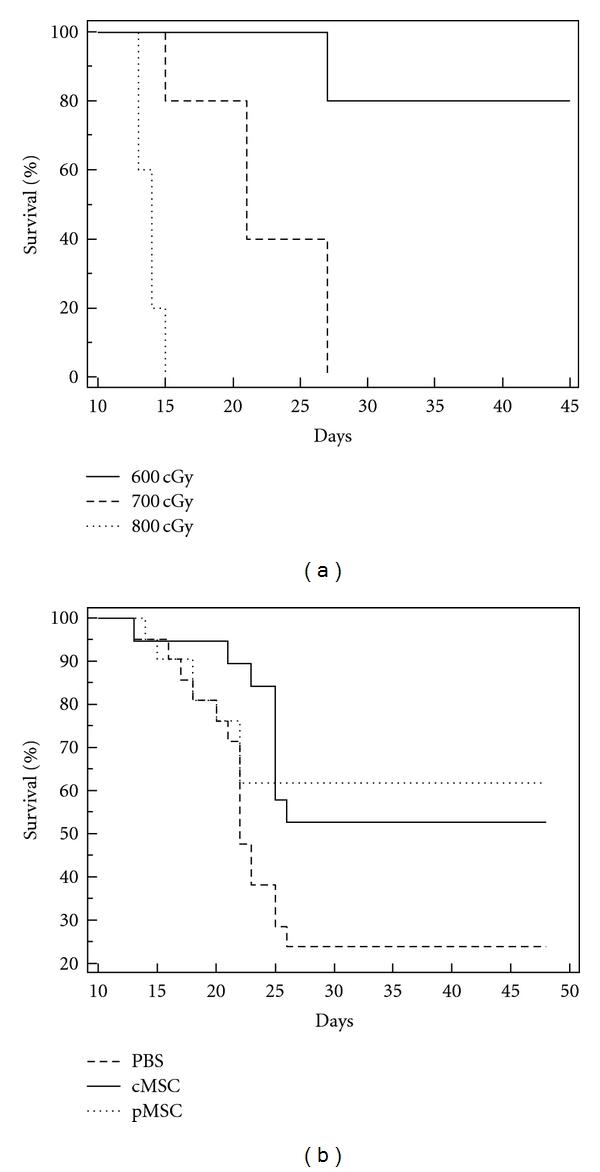
Impact of MSC infusion on survival of C57/Bl6 mice. (a) Determination of lethal dose of radiation for C57/Bl6 mice when radiation delivered with the RS2000 X-ray irradiator. Adult female mice 6 to 8 weeks in age were radiated with 600, 700, or 800 cGy radiation (*n* = 5 each) in two divided doses survival rate calculated at 7 weeks after radiation. None of the animals that received 700 or 800 cGy radiation survived past 4 weeks from day of radiation. 4 of the 5 mice in the 600 cGy radiation group survived past 7 weeks from day of radiation (*P* < 0.001). (b) Survival of mice receiving MSCs (clones or primary) when compared to control animals. Survival was studied in adult female mice receiving 700 cGy radiation followed by one of the following interventions: cloned MSCs (cMSC, *n* = 19), primary MSCs (pMSC, *n* = 20) or PBS (*n* = 21). Survival was significantly better for the cMSC (53%) and pMSC (60%) groups when compared to PBS (*P* values of 0.017 and 0.041, resp. with logrank test) at 7 weeks after radiation.

 Both the cloned MSC cell lines and primary MSCs were characterized for their ability to differentiate to adipogenic, chondrogenic, and osteogenic lineage by appropriate differentiation assays (Supplementary Figure 1). To assess the survival benefit of MSC infusion after hematopoietic injury from ionizing radiation, we first determined a suitable dose of radiation, which is lethal to most of the recipients when an X-ray irradiator (RS2000) was used. By administering increasing doses of radiation (starting at 600 cGy and increasing in increments of 100 cGy), it was determined that there was almost universal fatality at doses 700 cGy and higher when no specific therapeutic intervention was performed other than supportive care. Hence studies to determine efficacy of MSC infusion were performed at 700 cGy dose (Figure 1(a)). Mice that received 700 cGy of radiation were then treated with one of the following interventions: (1) single infusion of clonal MSC cells (mixture of 5 MSC cell lines in equal ratio, hence forth referred to as cMSC at total dose of 1 × 10^6^ cells, *n* = 19) (2) primary MSC (p MSC, at a dose of 1 × 10^6^ cells, *n* = 20), or (3) 100 *μ*L of PBS (*n* = 21). As shown in Figure 1(b), animals receiving either cMSC or pMSC had significantly improved survival when compared to the control PBS only group (*P* = 0.017 and 0.041, resp.) at the end of 7 weeks after radiation and cell infusion.

### 3.2. MSCs Are Rapidly Cleared after Infusion

**Figure 2:**
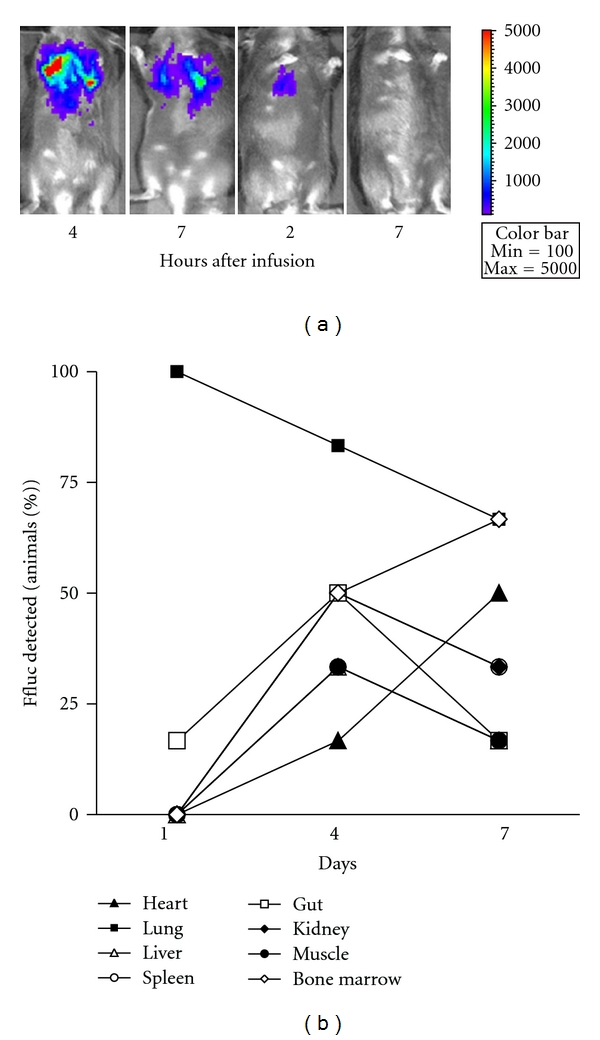
Tissue distribution of MSCs in vivo after injection. A. Bioluminescent imaging (BLI) of MSCs at 4, 7, and 24 hours after injection. 1 × 10^6^ MSC cells stably expressing ffluc were infused into recipient mice by tail vein injection followed by Bioluminescent signals were restricted to the thoracic area. Signals decreased rapidly and were undetectable 72 hours after infusion. B. Quantitative RT-PCR for ffluc in the above tissues harvested at 1, 4, and 7 days after infusion (*n* = 6 each). Whole RNA was prepared from tissues and analyzed for presence of ffluc mRNA by q RT-PCR. Since ffluc expression cannot be normalized to any endogenous control, equal quantities of starting material (2 *μ*g of whole RNA) were used for cDNA synthesis. Those samples with mean Ct < 35 cycles were deemed as positive for ffluc expression. All animals had detectable levels of ffluc mRNA in their lungs on day 1, while most other tissues had no detectable transcript on day 1. On days 4 and 7, there were detectable levels of ffluc transcripts detectable in a variable proportion of all tissues from some animals.

Although MSCs have shown promise to aid tissue repair in several clinical scenarios of tissue injury, the mechanistic basis of this beneficial effect remains unclear. Whole-organism imaging modalities including radio-isotype labeling [18–20], magnetic resonance imaging (MRI) [21, 22], and *in vivo* bioluminescence imaging (BLI) [23–25] have been used to determine the spatial and temporal distribution patterns of infused MSCs in different organs. These studies have generally shown that most of the infused cells are trapped in the lungs after systemic intravenous infusion and a small proportion is detectable in the target organs at various later time points. We hence attempted to determine the spatial distribution and kinetics of cMSC after infusion by three separate techniques. The five MSC cell lines were engineered to stably express firefly luciferase (ffluc) by use of lentiviral vectors. We then used bioluminescent imaging after intraperitoneal injection of luciferin substrate to determine tissue distribution of MSCs at specific time points after infusion. As shown in Figure 2(a), strong bioluminescent signals were detected from the chest region starting at 4 hours after infusion. The BLI signals rapidly decreased during the first 24 hours, and no bioluminescent signal was detected at 3 days after infusion. BLI signals were also not detected in any organ outside the thorax at any time point. Given that at least a few thousand cells expressing ffluc are necessary for unequivocal photon signals for BLI, we then attempted to determine the presence of ffluc expressing cells by both immune histochemistry (IHC) and polymerase chain reactions. Ffluc could not be reliably detected in any tissues including lungs by IHC at any time point presumably again due to their distribution across the entire pulmonary vasculature and not as discrete clumps of cells. Using reverse transcription and quantitative PCR, we then analyzed RNA from multiple tissues at three time points after infusion (days 1, 4, and 7) for presence of ffluc mRNA. As shown in Figure 2(b), ffluc was detectable in all animals one day after MSC infusion in the lungs, but mostly undetectable in any other tissue. On days 4 and 7, mRNA were detectable in other tissues also. Interestingly, cardiac and gut tissues showed a progressive increase in the proportion of animals with detectable ffluc transcript. Detection of transcripts using highly sensitive RT-PCR in the absence of BLI or IHC positivity suggests that the signals might arise from unviable cells or cellular debris that redistributed from the lung or from circulating RNA in peripheral blood. Together, these results suggest that most MSCs are passively filtered by the lung and few viable cells are redistributed from the lungs to other tissues after systemic infusion.

In summary, our results show that a single infusion of syngeneic marrow stromal cells (either immortalized cell lines or primary cells) can significantly improve hematopoietic recovery after lethal ionizing radiation in the murine model. These results are in agreement with another recent report from Lange et al. which also reported improved survival of lethally irradiated mice when MSCs are infused [12]. Although concomitant determination of hematopoietic recovery by sampling of peripheral blood at different time points after radiation and MSC infusion would have added further significance to these results, our pilot experiments revealed high mortality from repeated blood sampling in these animals (data not shown) and hence this experimental methodology was abandoned. Given that survival from similar dose range of ionizing radiation has been well documented to depend on hematopoietic recovery [1, 2]; survival alone was felt to be a robust and sufficient end point to study. Results from our study extend these observations by Lange et al. [12] to show that the infused cells are cleared rapidly by filtration in the lungs with little redistribution to other tissues including bone marrow. Consequently, the benefit of MSC infusion is likely to be indirect, mediated through release of soluble factors either from the MSC themselves or through other cells such as monocytes/macrophages, which interact with the MSCs in the lung. Although MSC infusion is clearly beneficial following ionizing radiation, the mechanistic basis of MSC-induced regeneration in various tissues including hematopoietic tissues remains unknown. Understanding the biological pathways would help to further improve efficacy of MSC-based therapeutics.

## Supplementary Material

Supplementary Material: This includes supplementary methods for differentiation of MSC to different lineages, production of lentiviral vectors and transfection of MSC, detailed methodology for bioluminescent imaging, and RNA extraction and quantitative RT PCR. Supplementary Figure 1 shows differentiation of MSC along adipocytic, chondrocytic and osteoid lineages.
