# Supplementary figures and images for: Marrow Stromal Cell Infusion Rescues Hematopoiesis in Lethally Irradiated Mice despite Rapid Clearance after Infusion
Source: Adv Hematol. 2012 Feb 16;2012:142530. doi: 10.1155/2012/142530 (PMC3287024; doi:10.1155/2012/142530)

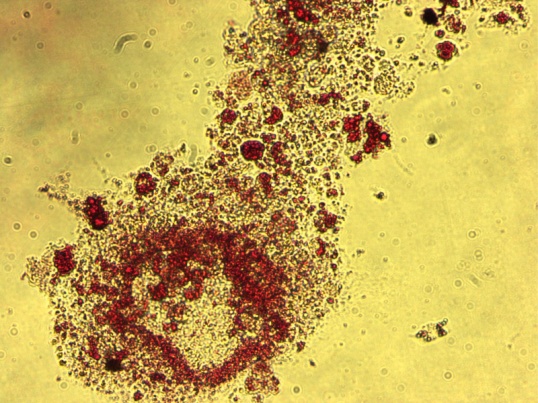

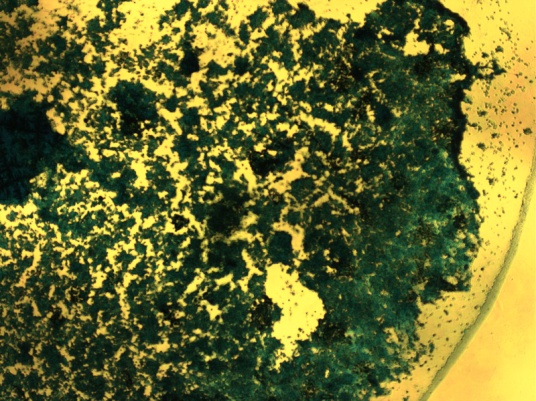

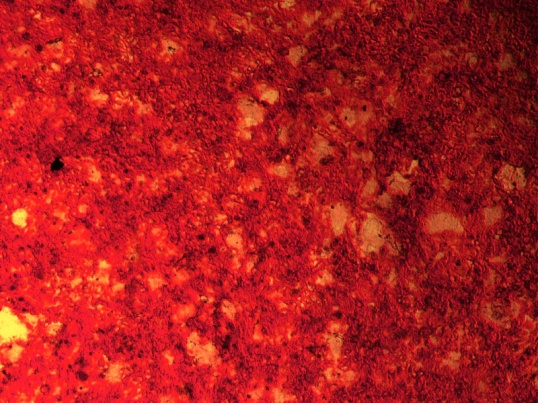


**A**

**B**

**C**

Supplement: Supplementary file 1 — Supplementary Material: This includes supplementary methods for differentiation of MSC to different lineages, production of lentiviral vectors and transfection of MSC, detailed methodology for bioluminescent imaging, and RNA extraction and quantitative RT PCR. Supplementary Figure 1 shows differentiation of MSC along adipocytic, chondrocytic and osteoid lineages. [file 142530.f1.docx]
